# Influence of Bilberry Pomace Powder Addition on the Physicochemical, Functional, Rheological, and Sensory Properties of Stirred Yogurt

**DOI:** 10.3390/gels10100616

**Published:** 2024-09-25

**Authors:** Ana Maria Blejan, Violeta Nour, Alexandru Radu Corbu, Georgiana Gabriela Codină

**Affiliations:** 1 Faculty of Food Science and Engineering, Dunărea de Jos University of Galati, Domnească Street 111, 800201 Galati, Romania; ana.blejan@ugal.ro; 2 Department of Horticulture & Food Science, University of Craiova, 13 AI Cuza Street, 200585 Craiova, Romania; corbu_lx@yahoo.co.uk; 3 Faculty of Food Engineering, Stefan cel Mare University of Suceava, 720229 Suceava, Romania; codina@fia.usv.ro

**Keywords:** bilberry pomace, stirred yogurt, gel, rheology, texture, phenolic compounds

## Abstract

Fruit processing by-products could represent a sustainable ingredient for developing innovative dairy products. The present study was conducted to develop a novel functional yogurt by adding bilberry pomace powder (BPP) at 0.5%, 1.0%, and 1.5% (*w*/*w*) levels in stirred-type yogurt production to confer color and to increase the dietary fiber and polyphenol content. Physicochemical properties of the yogurt samples, including color parameters, titratable acidity, pH, water holding capacity (WHC), and syneresis, as well as textural and rheological properties, were evaluated in yogurts on the 1, 14, and 28 days of refrigerated storage (4 °C). In addition, total phenolic content, total anthocyanin content, and radical scavenging activity were determined in yogurts, and sensory analysis was conducted. The results showed that BPP is a valuable source of polyphenols, dietary fiber, and oils rich in n-3 polyunsaturated fatty acids (n-3 PUFAs, n-6/n-3 ratio = 0.91). The incorporation of BPP imparted an attractive purple color to the yogurts, increased WHC, and reduced syneresis. Moreover, the addition of BPP improved the rheological properties, demonstrating that a more dense and stable yogurt gel network structure was obtained than the control. The yogurt enriched with 1.0% BPP received the highest scores for color, consistency, taste, and overall acceptability. Hence, bilberry pomace powder might be used as an ingredient to improve the nutritional and functional value of yogurts.

## 1. Introduction

The demand for novel functional food products formulated with natural components challenges the dairy industry to seek innovative ingredients. The fruit-processing by-products could represent a sustainable alternative as they are extremely valuable in terms of protein, lipid, micronutrients, bioactive substances, and dietary fiber content [1,2,3]. More than 190 million tons of by-products are generated in the global food industry each year [4] as a result of fruit and vegetable processing, including spoiled raw materials, peels, pits, seeds, oilseed cakes, and pomaces. They need to be disposed of appropriately through proper recycling and management; however, they can be profitably recovered. The processing and recovery of by-products for producing new food products can significantly contribute to reducing environmental impacts and promoting sustainable development [5].

Nowadays, bilberries are of significant interest and are often used as functional food ingredients, given their health benefits and various food, pharmaceutical, and nutraceutical applications. Bilberries (*Vaccinium myrtillus* L.) are currently harvested from wild bushes or cultivated in eastern Europe and northern Africa [6]. These fruits are recognized as being a valuable source of phenolic compounds and carotenoids, as well as containing moderate levels of vitamins and minerals. The high content of anthocyanins has also highlighted these fruits as interesting sources of coloring compounds for food applications. Delphinidin, cyanidin, petunidin, peonidin, and malvidin are the main aglycones in the bilberry anthocyanin profile, glycosylated with galactose, glucose, and arabinose. Numerous health benefits have been linked to the consumption of bilberries [6]. Karlsen et al. [7] found that bilberry juice decreased plasma markers of inflammation in subjects with an increased risk of cardiovascular disease, while Alnajjar et al. [8] reported that the consumption of bilberry extract may improve type 2 diabetes by lowering blood sugar levels.

The processing of bilberries in juice results in a by-product in the form of a pomace, consisting mainly of seeds and peels, which is rich in natural colorants, dietary fibers, polyphenols, and other antioxidant compounds [9]. At present, the bilberry pomace is discarded as waste, but it deserves to be saved and used to obtain new value-added products for the food, cosmetic, or pharmaceutical industries. For instance, Syrpas et al. [10] proposed the valorization of bilberry pomace by enzyme-assisted extraction, while Zhou et al. [11] proposed the employment of microwave hydrolysis and extraction to achieve the recovery of anthocyanins, saccharides, proteins, and inorganic salts from bilberry pomace. Despite their potential, most of these by-products remain underutilized for human consumption.

Recognized for its nutritional value and health benefits, yogurt is the most popular fermented milk product in the world [12]. The yogurt fermentation process involves the activity of two lactic acid bacteria: *Streptococcus thermophilus* and *Lactobacillus delbrueckii* subsp. *bulgaricus*. Numerous studies have shown that regular consumption of yogurt containing live cultures and probiotic strains lowers serum cholesterol levels and helps those suffering from lactose intolerance. According to Farvin et al. [13], the antioxidant activity of yogurt can be attributed to the antioxidant peptides issued by lactic acid bacteria during the fermentation process. Yogurt can relieve intestinal infections, inflammation, diarrhea, and colon cancer, among other conditions [14].

The health benefits of yogurt can be further enhanced by the addition of dietary fiber and bioactive/antioxidant compound sources [14]. The incorporation of fruits into yogurt adds extra value in terms of antioxidant properties and improves its sweetness and texture, making yogurt more flavorful and more enjoyable to eat [15]. The antioxidants from fruits, such as carotenoids, flavonoids, anthocyanins, and other polyphenols, can help prevent cell damage caused by free radicals, thus contributing to reducing the incidence of chronic diseases. In addition, fruits contain a variety of prebiotic fibers, which have been shown to promote digestive health, improve glucose tolerance in diabetics, and protect the body against the development of insulin resistance associated with type 2 diabetes [16].

Several studies have proposed increasing the functionality of yogurts by adding fruit by-products. Brahmi et al. [17] investigated the fortification of yogurts with apple peels and grape seed powder; Zahid et al. [18] reported on the nutritional capacity of freeze-dried mango peel powder as a source of antioxidant compounds and alpha-glucosidase inhibitors in probiotic yogurts. Additionally, de Toledo et al. [19] evaluated the potential of passion fruit peel and seed flour as a source of fiber and minerals to enhance the functional properties of stirred yogurt. Other previous studies also demonstrated that apple, banana, or passion fruit processing by-products [20,21], cactus pear peel [22], pineapple peel powder [23], or cranberry pomace [24] could be used as food ingredients with functional activity for yogurt production.

The present study was carried out to develop a novel functional product by enriching the stirred-type yogurt with bilberry pomace powder as a source of bioactive compounds and dietary fiber. Therefore, the main goals were to determine the nutritional and antioxidant content of bilberry pomace powder and to evaluate the effects of its addition at 0.5%, 1.0%, and 1.5% levels on the physicochemical, rheological, functional, and sensory properties of stirred-type yogurt during 28 days of refrigerated storage.

## 2. Results and Discussion

### 2.1. Proximate Composition, Titratable Acidity, and Fatty Acid Profile of Bilberry Pomace

Table 1 shows the proximate composition, titratable acidity, and fatty acid profile of bilberry pomace powder. According to the results, crude fiber was the major component (11.84 ± 0.48 g/100 g), followed by fat (8.21 ± 0.23 g/100 g) and protein (8.13 ± 0.34 g/100 g). Protein, fat, and minerals in BPP could enhance the nutritional value of the yogurt, while the high fiber content makes BPP a good ingredient in the formulations of yogurt supplemented with dietary fiber.

Linolenic acid (C18:3n-3) was the dominant fatty acid in the oil of BPP (37.81%), in agreement with Pires et al. [25] reporting 32.9% linolenic acid in dried bilberry fruits and Yang et al. [26] who found 36.1% linolenic acid in bilberry seed oil. By contrast, Dulf et al. [27] reported only 18.7% linolenic acid in wild blueberry pomace. The fatty acid composition of BPP comprised 72.86% PUFA, 18.64% MUFA, and 8.21% SFA, in line with the fatty acid profile previously reported by Pires et al. [25] in dried bilberry fruits (75.3% PUFA, 16% MUFA, and 8.8% SFA). A low n-6/n-3 ratio of 0.91 was found in BPP in the present study. In line with our result, Yang et al. [26] reported an n-6/n-3 ratio close to 1:1 in bilberry seed oil, while Van Hoed et al. [28] reported an n-6/n-3 ratio = 1.50 in blueberry seed oil. Numerous studies demonstrated that a high dietary n-6/n-3 ratio contributes to the development of many chronic inflammatory diseases like cardiovascular diseases and cancers [29]. Moreover, reducing the n-6/n-3 ratio is suggested as beneficial for human health in connection with the anti-inflammatory effects of n-3 PUFAs, especially alpha-linolenic acid, and longer-chain n-3 polyunsaturated fatty acids [30]. The high content of PUFAs and the low n-6/n-3 ratio make BPP a promising natural ingredient for developing new food products with improved fatty acid profiles. A total phenolic content of 37.22 ± 0.56 mg GAE/g and a total anthocyanin content of 28.66 ± 0.46 mg GCE/g were found in BPP in the present study. In a previous study, our research team found that the phenolic profile of the bilberry pomace powder was dominated by ellagic acid (13.60 mg/g), followed by catechin (10.86 mg/g), and procyanidin dimer B1 (3.91 mg/g). Quercetin (2.52 mg/g), protocatechuic acid (1.91 mg/g), and epicatechin (0.85 mg/g) were also quantified in bilberry pomace powder [31].

### 2.2. Color

Yogurt color, as the first attribute perceived by consumers, has a great impact on their preferences. Table 2 shows the evolution of the color parameters (L*, a*, b*), C* (chroma), h* (hue angle), and ΔE of control and enriched yogurt samples at 1, 14, and 28 days of storage. Regarding the L* coordinate, which indicates the lightness, it decreased more as the level of BPP addition increased. A similar gradual reduction in the L* values was previously reported by Dinkçi et al. [32] as a result of the progressive hazelnut skin addition in functional yogurt or by Cho et al. [33] and Wang et al. [34] in yogurt enriched with green olive powder and apple pomace powder, respectively. A progressive decrease was also noticed for b* values, accompanied by the gradual increase in a* values as the level of BPP addition increased as a response to the increase in the anthocyanin content. The b* values were negative, in line with those reported by Szołtysik et al. [35] in yogurts enriched with blue honeysuckle berries dry polyphenolic extract. Color intensity (chroma, C*) increased as the concentration of bilberry pomace powder increased, shifting towards purple. This fact is related to the dark purple color of bilberry pomace powder given by its high anthocyanin content coming from the skins and from the residual pulp of bilberries, which acted as a natural colorant in yogurts. As expected, the ΔE value was highest for the yogurts enriched with 1.5% BPP.

**Figure 1:**
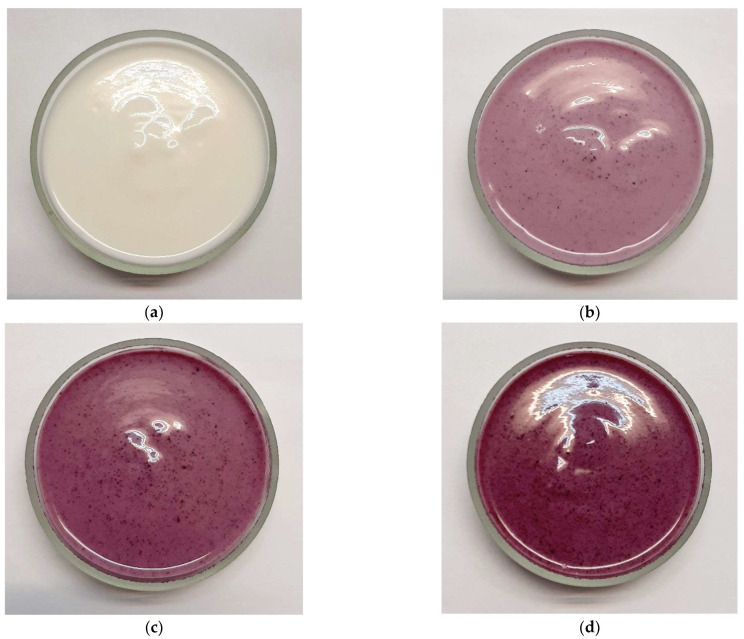
The appearance of the yogurt samples: (**a**) YC—control yogurt; (**b**) Y0.5%BPP—yogurt with 0.5% BPP addition; (**c**) Y1.0%BPP—yogurt with 1.0% BPP addition; (**d**) Y1.5%BPP—yogurt with 1.5% BPP addition.

The yogurts enriched with BPP presented an attractive purple color, favored once more by their pH values located in the acidic range (Figure 1). During storage, a* values had a significant decreasing trend in the supplemented yogurt samples, while in control yogurt, the variations of L* and a* values were not significant. A significant (*p* < 0.05) increase in the b* values was recorded during storage in the supplemented yogurt samples, while in the controls, b* values significantly (*p* < 0.05) decreased. As a result, chroma (C*) significantly (*p* < 0.05) decreased during storage in all enriched formulations. The decreasing trend of a* values, accompanied by the increasing trend of b* values during storage, could be attributed to the degradation of anthocyanins, which are important natural pigments in bilberry pomace. Ścibisz and Ziarno [36] also reported on the accelerated degradation of anthocyanins in yogurt-containing strawberry, raspberry, and blueberry smoothies during storage and attributed this behavior to the high hydrogen peroxide content and to the enzymatic activity of the lactic acid bacteria in yogurt. Cheng et al. [37] evaluated the color stability and degradation of anthocyanins in mulberry-stirred yogurt and found that the redness value significantly decreased during storage, as well as the anthocyanin content and the degradation kinetics following a first-order reaction. Hydrogen peroxide, resulting from the lactic acid bacteria activity, is a reactive oxygen species able to facilitate the breakdown of anthocyanins. Moreover, some enzymes produced during lactic fermentation, such as β-glucosidase, proved to be able to hydrolyze the glycosidic bonds of anthocyanins, thereby altering the color [38,39]. The color differences between samples and controls (ΔE) differed significantly (*p* < 0.05) in all three sampling times.

### 2.3. Physicochemical Properties

Table 3 presents changes in pH and acidity of control and enriched yogurt samples during the storage period. Both enrichment with bilberry pomace powder and storage time significantly influenced (*p* < 0.05) the pH and acidity values of yogurts. pH level significantly decreased (*p* < 0.05) in response to the addition of bilberry pomace powder by as much as the level of addition was higher due to the high acidity of BPP (5.45 ± 0.31 g citric acid/100 g, Table 1). pH of all yogurt samples decreased during the storage period while acidity increased. Similar evolutions have been noticed previously in yogurt supplemented with various dry plant preparations such as wine grape pomace powder, blueberry flower pulp, apple pomace powder, and blue honeysuckle berries dry polyphenolic extract and have been attributed to the continuous production of lactic acid during the lactic fermentation by the lactic acid bacteria [34,35,40,41].

Syneresis (%), the percentage of serum released from the yogurt gel matrix, is a critical parameter for yogurt, which affects consumer acceptance [42]. A higher level of syneresis is indicative of a lower quality [43]. Syneresis is mainly found in set yogurt, which is considered a textural defect, but stirred yogurts are also prone to it, mainly regarding the loss of gel rigidity [42]. Water holding capacity in gel networks is one of the main influencing factors determining yogurt’s stability against syneresis; a higher WRC will determine the decrease in susceptibility to syneresis during storage [44].

The enriched yogurt samples were analyzed for their syneresis and water retention capacity at days 1, 14, and 28 of storage, and the results are shown in Table 3. On the first day, the highest value of syneresis was found for control yogurt (59.99 ± 0.34%). The addition of BPP determined a significant decrease (*p* < 0.05) of syneresis as compared with the control yogurt at all sampling times, which can be assigned to the capacity of the bilberry pomace powder to absorb the whey released by the gel structure, which in turn is determined by the high fiber content of BPP. Previous studies also found that syneresis was reduced by fiber addition due to their ability to interact through hydrogen bridges with charge moieties on the surface of casein and to increase the compactness and stability of the protein network, thus immobilizing a larger amount of free serum [45,46]. Bilberry dietary fiber carbohydrate is composed of pectin, hemicellulose, and cellulose [47]. Previous studies on cell wall carbohydrates in bilberries found pectin contents varying between 0.10 and 0.78 g/100 g [48]. In juice processing with only pressing without adding pectinolytic enzymes, juice yield from bilberries is very low because a highly viscous pectin gel is formed after mashing [48]. Pectin from BPP is capable of interacting with positive charges on the surface of the proteins through calcium ions, strengthening the protein network and preventing aggregation, sedimentation, and separation of whey through ionic and steric stabilization effects [43]. Lowering the pH through the addition of BPP could also contribute to reducing syneresis because the pH moves away from the isoelectric point of casein (pH~4.6), at which the electrostatic repulsion is minimal. Ahmed et al. [49] also reported that syneresis tended to decrease with the addition of apple pomace and pomegranate peel powders, while Dinkçi et al. [32] observed the same evolution as a result of yogurt enrichment with hazelnut skin. WHC increased gradually with increasing BPP addition level in enriched yogurt samples; maximum WHC was found in Y1.5%BPP at day 1 of storage (49.24 ± 1.31%), which was 11% higher than that of control yogurt (44.80%). Syneresis increased significantly (*p* < 0.05) during storage both in the control and enriched yogurt samples. At present, it is generally accepted that the increased syneresis with storage time is associated with severe reorganization of the casein network through enhancement in the particle–particle junction that leads initially to micro-localized syneresis. Further, these localized rearrangements result in the formation of serum channels and pouches, which promote the expulsion of the interlocked whey [42,50,51].

### 2.4. Texture Analysis

The results on the instrumental texture parameters (hardness, adhesiveness, gumminess, stickiness, chewiness, and cohesiveness) of the yogurts are shown in Table 4. A significant effect of the BPP addition was observed for all textural parameters. The values of hardness, cohesiveness, and gumminess significantly increased (*p* < 0.05) as the amount of BPP addition increased, which was maintained throughout the storage period. These results could be attributed to the increment of the total solid, fiber, and phenolic content in yogurt resulting from BPP addition. The firmness of yogurt is directly related to its total solid content [52]. Amal et al. [53] also reported that a lower moisture content and a higher total solid content determined the increase in firmness and consistency of yogurt.

Pan et al. [54] used pomegranate juice powder as a sugar replacer in yogurt and found that the higher the pomegranate juice powder level, the firmer in texture and compacter in microstructure the yogurt became. They attributed this behavior to the phenol–protein interaction as a result of the strong affinity for caseins of phenolic hydroxyl groups [55]. In good agreement with our results, Oroian et al. [56] reported that hardness, chewiness, and gumminess increased while adhesivity and stickiness of the yogurt samples decreased as a result of cranberry powder addition and attributed this evolution to the pectin content of cranberry powder. Wang et al. [34] reported that the addition of apple pomace in yogurt at 3% doubled firmness and consistency and more than doubled the cohesiveness and viscosity index, while Karnopp et al. [57] found that the addition of grape skin flour increased the hardness and consistency of yogurt. The increase in cohesiveness was ascribed to the high viscosity and whey absorption capacity of apple pomace or grape skin flour, largely assigned to their high dietary fiber content, which is capable of reducing syneresis and strengthening the internal protein gel structure. However, Dinkçi et al. [32] found that hardness and consistency values in yogurts significantly decreased as the amount of hazelnut skin increased at a 2–3% enrichment level. The addition of 1.5% pineapple peel powder also resulted in lower hardness of yogurt [23]. In this case, the gel weakening has been attributed to the thermodynamic incompatibility between milk proteins and polysaccharides from pineapple peel powder. The increase in gumminess and the decrease in adhesiveness were also previously reported in yogurt enriched with cellulose fiber [58] or dried grape pomace [59], and they were mainly attributed to the increment of fiber concentration.

Adhesiveness and stickiness significantly decreased during storage in all samples, while the values of hardness, gumminess, and chewiness increased in the first week of storage, exhibited the maximum values on day 7th, and decreased in the second week of storage. Pan et al. [54] also found an increase in firmness and consistency during storage both in control yogurts and in those supplemented with pomegranate juice powder as a sugar replacer, while Postolache et al. [60] reported a decrease in adhesiveness during storage of yogurt fortified with Rhododendron flower powder as a functional ingredient.

### 2.5. Rheological Properties

**Figure 2:**
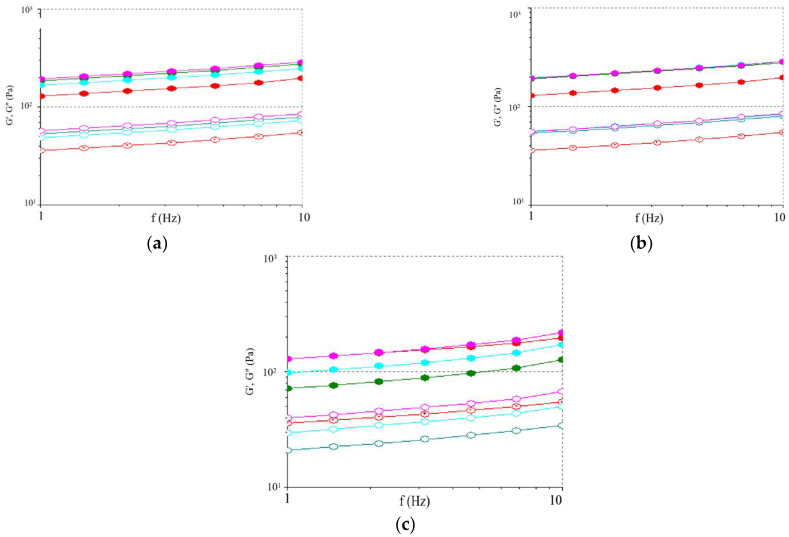
Evolution of the storage modulus (G′—solid symbols) and loss modulus (G″—open symbols) of the yogurt samples during the storage period: (**a**) 1 day of storage; (**b**) 14 days of storage; (**c**) 28 days of storage; -•- YC—control yogurt; -•- Y0.5%BPP—yogurt with 0.5% BPP addition; -•- Y1.0%BPP—yogurt with 1.0% BPP addition; -•- Y1.5%BPP—yogurt with 1.5% BPP addition.

The yogurt’s rheological properties offer useful information on its viscoelastic behavior over the frequency range of 1 to 10 Hz. The storage modulus (G′) and the loss modulus (G″) indicate the elastic (solid-like) and viscous (liquid-like) behavior, respectively [45]. The evolution of G′ and G″ values of yogurt samples as a function of frequency is shown in Figure 2a–c.

According to our data, the G″ values were lower than G′ values for the entire frequency range, which showed a predominantly elastic behavior for all yogurt samples. The G′ and G″ values were higher in the BPP-enriched yogurt samples in comparison to the control sample. This behavior could be attributed to the interaction between the dietary fibers from BPP and yogurt proteins (mainly caseins), thus establishing a more solid network structure. The present results are in agreement with those reported by Liu and Lv [41], who concluded that the addition of blueberry flower pulp may lead to a more stable structure of yogurt. The G′ values are an indicator of the strength of the bonds in the casein network [24]. When BPP is added to yogurt, it is entrapped by the casein network, which becomes more compact, leading to higher G′ values. It seems that BPP provides a “filler” effect, partially due to its fiber content that stabilizes the serum phase in the casein network. According to previous studies, fiber addition in yogurt products affects their gel-like structure [23,45,61]. Varnaitė et al. [24] reported that yogurt microstructure presented numerous large pores evenly distributed in the protein matrix, whereas the microstructure of yogurt enriched with fiber-rich cranberry pomace showed a more compact protein matrix with smaller and fewer pores. A high number of pores in the yogurt microstructure is indicative of a weaker gel [62]. According to our data, the BPP addition led to a stronger gel network as compared with the control sample. The evolution of yogurt rheological properties during the 28-day storage period is shown in Table 5.

There was an increase in rheological values until the 14th day, followed by a decrease until the end of the storage period. According to Guénard-Lampron et al. [63], the increase in rheological values may be related to the post-acidification process. During initial storage, syneresis and flow resistance are relatively stable, which increases yogurt rheological values. Moreover, the BPP addition favored the reabsorption of the whey in the gel, which led to a more dense and stable protein network. All the yogurt samples with BPP addition presented higher rheological values with respect to the control sample. This may also be attributed to the polyphenols from BPP, which can bind to caseins to form protein–polyphenol complexes, resulting in higher viscosity and dynamic module values [64]. In addition, the dietary fibers from BPP could retain water, which may explain the high rheological values of the BPP-enriched yogurts. However, a prolonged storage period of yogurt led to a loss of the protein’s ability to hold water, affecting its gel structure and leading to a weaker gel network, reflected by the lower values of the viscoelastic parameters [23].

### 2.6. Total Phenolic Content, Total Anthocyanin Content, and DPPH Radical Scavenging Activity

The total phenolic content (TPC), total anthocyanin content (TAC), and DPPH radical scavenging activity (RSA) values of yogurt samples are presented in Table 6. The quantification of apparent phenolic content in the control sample, which obviously does not contain phenolic compounds, can be assigned to a limitation of the Folin–Ciocalteu method [65]. TPC and TAC were found to increase significantly (*p* < 0.05) as the BPP addition level raised in yogurt, which was to be expected considering the high TPC (37.22 ± 0.56 mg GAE/g) and TAC (28.66 ± 0.46 mg GCE/g) found in BPP. On the first day of storage, the highest TPC values were found in the yogurt enriched with 1.5% BPP (62.45 ± 1.66 mg GAE/100 g). Du et al. [66] reported similar TPC and TAC values in a functional-flavored yogurt supplemented with mulberry pomace. The addition of 1% BPP in our study increased by 4.28 times the total phenolic concentration in fortified yogurt. Increases in TPC only by 1.36, 1.61, and 1.82 times have been found previously by adding 1, 2, and 3% (*w*/*w*) apple pomace as a functional ingredient in stirred-type yogurt [34], while Marchiani et al. [67] reported increases of 37.3–68.7% in the total phenolic content in yogurt enriched with grape pomace obtained from different cultivars.

TPC, TAC, and RSA increased in the first 14 days of storage compared to their initial values, then significantly decreased (*p* < 0.05). Sahingil and Hayaloglu [68] also reported that the TPC of rosehip-supplemented yogurt increased gradually during storage, with the highest values reached at 15 days. Other researchers also reported that TPC and antioxidant activity values gradually increased during storage in yogurt enriched with hazelnut skins [69] or mulberry pomace [66]. They associated this evolution with an increase in the extractability of polyphenols and their gradual release from the complexes formed with milk proteins over time. The release of polyphenols may be influenced by the decrease in pH values, alteration of protein structure, or enzymatic and bacterial activity in yogurt [34,70]. However, Tseng and Zhao [40] and Cho et al. [33] reported a decreasing trend of TPC and antioxidant activity during storage in yogurt fortified with wine grape pomace and green olive powder, respectively. They attributed this temporary decrease in TPC in yogurt to the decomposition of polymeric phenolic compounds in the presence of lactic acid bacteria. The high RSA found in BPP in the present study (26.55 ± 0.64 μmol TE/g) confirms that the incorporation of BPP may be a suitable alternative for augmenting the antioxidant activity of yogurt and protecting against lipid oxidation. Augmenting the addition level of BPP from 0.5% to 1.5% significantly (*p* < 0.05) increased the antioxidant activity of the supplemented yogurt compared to the control (from 0.77 to 0.98 μmol Trolox/g). These findings are in line with those of Varnaitė et al. [24] and Ribeiro et al. [71], who found increased antioxidant activity in yogurt enriched with cranberry pomace and olive pomace powder, respectively, and attributed this increase to the high polyphenols content of these by-products. However, RSA increased only by 1.39 times for a 1% BPP addition level, while TPC increased by 4.28 times. The lower multiplication level of antioxidant activity in yogurt compared to that of TPC is due to the fact that control yogurt already has a high RSA due to its high content of antioxidant compounds, mainly bioactive low molecular weight peptides produced during lactic fermentation of milk. Other metabolites resulting from fermentation, such as free amino acids, fatty acids, folate, or enzymes, have oxidation–reduction potential, being able to enhance the antioxidant capacity of yogurt [72]. After 28 days of refrigerated storage, DPPH radical scavenging activity of Y0.5%BPP, Y1.0%BPP, and Y1.5%BPP yogurts decreased by 7.79%, 6.97%, and 10.20%, respectively. Decomposition of phenolics by polyphenol oxidase of lactic acid bacteria and protein–polyphenol interaction are probably the main reasons for the decreasing trend of the radical scavenging activity in the supplemented yogurts during storage, as it is generally known that phenolic compounds contribute significantly to the antioxidant activity [40,47,54,55,66].

### 2.7. Sensory Evaluation

**Figure 3:**
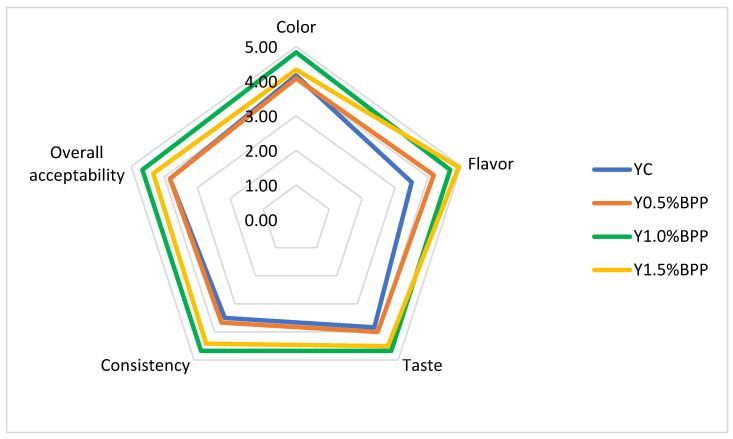
Sensory evaluation of the control and enriched stirred-type yogurts.

The sensory evaluation of the control and newly developed functional yogurts was conducted on the basis of 24 responses. The mean scores recorded for color, flavor, taste, consistency, and overall acceptability of yogurts are shown in Figure 3. Statistical analysis reveals that there were significant differences (*p* < 0.05) between the control and the enriched yogurts for all sensory attributes.

The most noticeable change induced by the addition of BPP is that of the color, which became purple: the higher the BPP addition level was, the deeper the purple color was (Figure 1). Bilberry pomace has a very high content of anthocyanins, mainly from the skins, and the acidic pH of the yogurt allows the development of a delightful and very attractive purple color. Sahingil and Hayaloglu [68] also found that the addition of rosehip pulp showed a considerable positive impact on the yogurt color. The highest scores for color were obtained by the Y1.0%BPP formulation, followed by Y1.5%BPP, which was dark purple, and Y0.5%BPP, which had a pale purple color. The bilberry aroma was strong in the yogurt with 1.5% BPP addition, which was the best rated for flavor, followed by the Y1.0%BPP formulation. The taste improved in all yogurt samples as a result of BPP addition as compared to the control, as a slightly sour and sweet taste was added to yogurt by enrichment, while the special and unique bilberry taste and aroma of the novel yogurts delighted the palates of the panelists. Similarly, Liu and Lv [41] found that aroma, taste, and texture acceptance scores of yogurt enriched with 2–5% blueberry flower pulp were higher than those of the control. The consistency also improved after the addition of 0.5% and 1% BPP as the texture became firmer, which was well appreciated by the panelists. However, at the 1.5% BPP addition level, the consistency and viscosity of the stirred-type yogurt increased quite a lot, and its fluidity decreased. As fluidity is an important feature of a stirred-type yogurt, a lower mean score was received for consistency by this formulation. As for the overall acceptability, the best-rated formulation was Y1.0%BPP, with the highest mean score of 4.67 on the rating scale. 

**Figure 4:**
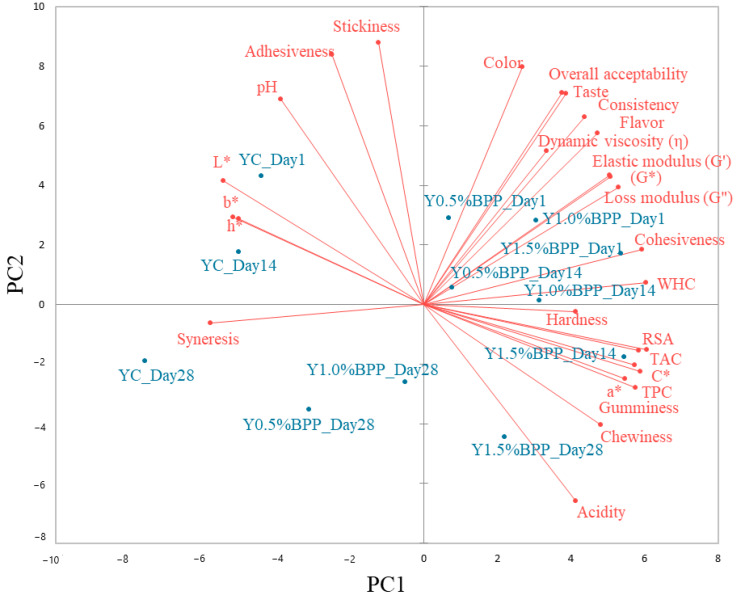
Principal component analysis of the yogurt samples during 28 days of storage and their physicochemical, functional, rheological, and sensory characteristics: YC—control yogurt; Y0.5%BPP—yogurt with 0.5% BPP addition; Y1.0%BPP—yogurt with 1.0% BPP addition; Y1.5%BPP—yogurt with 1.5% BPP addition; L*, a*, b*, C*, h*—color parameters; WHC—water holding capacity; G*—complex modulus; TAC—total antocyanic content; TPC—total phenolic content; RSA—DPPH radical scavenging activity.

The relationships between the characteristics of yogurt samples were examined by principal component analysis (PCA), and the results are shown in Figure 4. The PC components explain 87.26% of the total variance (PC1 = 60.40% and PC2 = 26.86%). According to the PCA graph, positive correlations were found between textural parameters adhesiveness and stickiness (r = 0.90), color parameters L*, b*, and h* (r = 0.92, r = 0.91), pH, and adhesiveness (r = 0.93), which are placed on the top left of the graph. Positive correlations between yogurt color parameters L*, b*, and h* have also been reported by Yilmaz-Ersan et al. [73]. Adhesiveness is a textural parameter that offers information on the product extension behavior and may be associated with the ropy character of yogurt. Yogurt pH strongly depends on its acidifying and post-acidifying properties. BPP addition decreased the pH values of the yogurt samples out of the linear zone (>4.45), a fact that correlates with the adhesiveness value [74]. BPP addition affects the color characteristics of yogurts, which placed the L*, b*, and h* values on the same part of the PCA graph with pH, adhesiveness, and stickiness values. Also, a close association has been obtained between all sensory characteristics, dynamic rheological modules, cohesiveness and loss modulus (r = 0.89), cohesiveness and water holding capacity (WHC) (r = 0.95), which were placed on the top right of the graph. Similar to our results, Janhøj et al. [75] reported significant correlations between dynamic rheological data of yogurts and their sensory and textural characteristics.

On the right bottom of the graph, there is a closeness between the total phenolic content, total anthocyanin content, and DPPH radical scavenging activity values of yogurt samples and also between these parameters and the color ones C* and a*. All these parameters are related to the antioxidant activity, whose values have increased with the increasing BPP levels. Also, the a* and C* values increased with the increasing level of BPP in yogurt due to the presence of anthocyanins [68], which gave a purple color to the yogurt samples. Along the PC1 and PC2 axes, negative significant correlations have been obtained between syneresis and color parameters a* (r = −0.91) and C* (r = −0.93), WHC (r = −0.89), cohesiveness (r = −0.88), dynamic modules, sensory characteristics, total phenolics content (r = −0.77), total anthocyanins content (r = −0.80), and radical scavenging activity (r = −0.84). A high syneresis will lead to a weak-bodied yogurt with a low gel strength and a grainy textured yogurt [76]. Moreover, consumers demand yogurt with a smooth viscous gel and a low whey separation, which agreed with the findings of Yilmaz-Ersan et al. [73] that sensory parameters of the yogurt samples were negatively correlated with cohesiveness and a* value. Also, acidity has been negatively correlated with pH (r = −0.97), adhesiveness (r = −0.91), and stickiness (r = −0.79). Generally, in terms of the first principal component, PC1, it may be seen that there is a close association between yogurt samples that have been related to their storage period. PC2 distinguishes an association between control yogurts and the samples with BPP addition on days 1 and 14 of storage. The yogurt samples from day 1 of storage are more related to the sensory characteristics and dynamic rheological values, indicating that they are the most preferred by consumers. The yogurt samples with the highest level of BPP addition in their recipe from days 14 and 28 of storage were mostly associated with the total phenolic content, total anthocyanin content, and DPPH radical scavenging activity values. This fact is explainable since BPP is a good source of phenolic compounds, especially anthocyanins, which contribute to the high levels of antioxidant activity. Moreover, protein hydrolysis occurred during storage, leading to the formation of small peptides, which will increase the antioxidant activity. Similar data have also been reported in previous studies [68,77].

## 3. Conclusions

The chemical composition showed that BPP is a valuable source of oils rich in polyunsaturated fatty acids and dietary fiber. Moreover, the results of the bioactive investigation demonstrated that BPP is a good source of polyphenols and anthocyanins with strong colorant properties and high antioxidant activity. BPP was successfully incorporated into yogurt at 0.5, 1.0, and 1.5% levels to impart an attractive range of purple color. Although it determined a slight increase in acidity, the enrichment with BPP significantly increased water-holding capacity and reduced syneresis in yogurt. The values of hardness, cohesiveness, and gumminess increased while adhesiveness and stickiness decreased with increasing BPP addition level, while the results of the rheological measurements demonstrated that a more dense and stable yogurt gel network structure was obtained as compared with the control. In addition to the nutritional and antioxidant benefits, the addition of BPP improved the sensory properties of yogurts. The yogurt made with a 1.0% BPP addition was found to be the most suitable in terms of color, consistency, taste, and overall acceptability. Consequently, this study demonstrated that bilberry pomace powder could be a good candidate as a sustainable ingredient for producing a novel functional stirred-type yogurt with improved bioactive, textural, and sensory properties to provide more diversity in yogurt choices to consumers.

## 4. Materials and Methods

### 4.1. Materials

**Figure 5:**
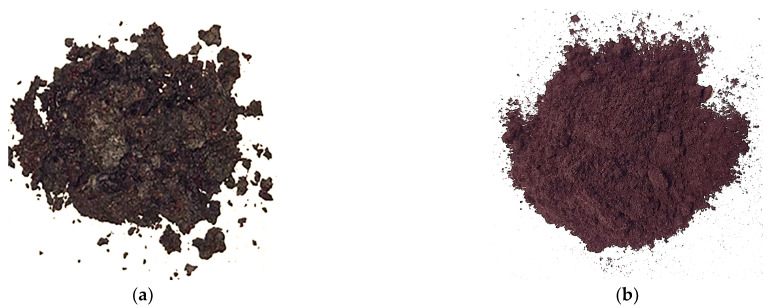
(**a**) Dried bilberry pomace; (**b**) bilberry pomace powder.

Wild bilberries (*Vaccinium myrtillus* L.) harvested during the 2023 harvest season from the wild flora of Valcea county (South-West Oltenia Region, Romania) were processed in juice at Jiancom S.R.L., an industrial fruit processing company in Vaideeni (Vâlcea county, South-West Romania, Romania). Two batches of 3 kg fresh bilberry pomaces, consisting of peels, seeds, and residual pulp, were collected as by-products of bilberry juice manufacture on different days just after the conventional juice processing without enzymatic treatment. They were packed in sealed plastic sacks, transferred to the lab, and dried at 57 °C in a Deca +SS Design laboratory dryer (Profimatic, Bucharest, Romania) until the moisture content was less than 10%. The dried pomaces were ground to make a powder to pass through a 0.50 mm sieve, packed in sealed glass pots, and stored in ambient conditions until further analysis and use. Before the yogurt preparation, the bilberry pomace powder was treated by UV light irradiation for 40 min. The raw form of dried bilberry pomace and powder is shown in Figure 5.

Fresh natural yogurt was purchased from a local market (Craiova, Romania). Nutrition facts of the yogurt are shown in Table 7.

### 4.2. Reagents

Trolox (6-hydroxy-2,5,7,8-tetramethylchroman-2-carboxylic acid), DPPH (2,2-diphenyl-1-picrylhydrazyl), gallic acid, Folin–Ciocalteu reagent, and sodium acetate were purchased from Sigma-Aldrich (Steinheim, Germany). Potassium chloride, anhydrous sodium carbonate, hydrochloric acid, and methanol were supplied by Merck (Darmstadt, Germany).

### 4.3. Preparation of Stirred Yogurt Samples

Four lots of yogurt were prepared: control yogurt without any addition (YC) and the other lots enriched with 0.5% (Y0.5%BPP), 1.0% (Y1.0%BPP), and 1.5% (Y1.5%BPP) bilberry pomace powder. After they were mixed gently (3 min) to incorporate the bilberry pomace powder, all yogurt samples were filled into 200 mL sterilized glass containers and stored at 4 °C in a fridge. Samples were collected on the 1, 14, and 28 days of storage for the physicochemical, rheological, and biochemical analyses. Two replicates of each batch were prepared and analyzed, and the sample analysis was repeated three times.

### 4.4. Proximate and Fatty Acid Composition of Bilberry Pomace Powder

The basic chemical composition, such as dry matter, protein, fat, fiber, and ash content of bilberry pomace powder, was examined according to standard methods [78]. The protein content was analyzed according to the Kjeldahl procedure using an automated nitrogen analyzer (UDK 149, Velp Scientific, Milan, Italy), while the fat content was assayed after extraction in organic solvents with a Soxhlet automatic extraction system (SER 148/3, Velp Scientific, Usmate, Italy). The ash content was determined using a Caloris CL 1206 oven (Bucharest, Romania), while the fiber content was determined by digestion with acid and alkali using an automatic analyzer (Fibertec 2010, Tecator, Hillerod, Sweden). The fatty acid profile was determined by gas chromatography of fatty acid methyl esters (FAME) as described by Blejan et al. [31], and the results were expressed as grams of fatty acid per 100 g of total fatty acids.

### 4.5. Color Analysis

The CIEL* a* b* color parameters [L*—lightness from black (0) to white (100), a*—color coordinate from green (−) to red (+), and b*—color coordinate from blue (−) to yellow (+)] of yogurt samples were measured with a PCE-CSM1 colorimeter (PCE Instruments, Southampton, UK) calibrated before measurements using a standard white plate. Five readings were performed at different locations on each sample. Chroma (C), indicating the color intensity, was calculated as (a*^2^ + b*^2^)^1/2,^ while hue angle (h), indicating color tonality, was calculated as arctan (b*/a*). Delta E (ΔE) was calculated to measure the changes in color of the enriched yogurts compared to the control yogurt using the formula below:

$$\Delta E=\sqrt{{\left(L_{1}-L_{0}\right)}^{2}+{\left(a_{1}-a_{0}\right)}^{2}+{\left(b_{1}-b_{0}\right)}^{2}}$$

where L_1_, a_1_, b_1_ represent color values of yogurt samples, while L_0_, a_0_, b_0_ are color values of control yogurt.

### 4.6. Titratable Acidity and pH

The titratable acidity was determined after titration with 0.1 N NaOH in the presence of 1% phenolphthalein and expressed as % lactic acid. Since the color change in yogurts enriched with bilberry pomace powder was not relevant, the titration was carried out until pH reached 8.1 [78]. The pH of yogurt samples was measured in triplicate at room temperature (20 °C) using a Hanna pH-meter HI255 (Hanna Instruments, Padova, Italy).

### 4.7. Syneresis and Water Holding Capacity

The syneresis of the yogurts was determined by measuring the volume of separated whey collected after 6 h of drainage from 100 mL of yogurt sample placed in a funnel lined with Whatman nr. 1 filter paper at 20 °C. Syneresis was calculated as follows [79]:

S = (V1/V2) × 100

where V1 is the volume of whey collected after drainage, and V2 is the volume of the yogurt sample.

The water-holding capacity (WHC) of yogurts was determined according to Barkallah et al. [79]. A sample of about 5 g of yogurt was centrifuged for 15 min at 4500× *g*. The resultant supernatant was removed and weighed. The water holding capacity was expressed as a percentage (%) and calculated as follows:

WHC (%) = (1 − W1/W2) × 100

where W1 is the weight of the whey separated after centrifugation, and W2 is the weight of the yogurt sample. All of the analyses were performed in triplicate.

### 4.8. Texture Analysis

The hardness (g), adhesiveness (J), gumminess (g), stickiness (g), chewiness (g), and cohesiveness (dimensionless) of yogurts were determined using the Perten TVT 6700 texturometer (Perten Instruments, Hägersten, Sweden) equipped with a 65 mm height and 35 mm diameter stainless steel compression plate and an 8 mm height back extrusion container holder. To obtain the texture profile, a multicycle compression test was performed. The sample height was 25 mm, and the starting distance from the sample was 10 mm. The device settings were as follows: resting time between the two compression cycles: 5 s, test speed: 1 mm/s, retract speed: 5 mm/s, trigger force: 10 g. The determinations were repeated five times for each individual sample.

### 4.9. Rheological Measurements

A Modular Advanced Rheometer System (Thermo Scientific, Thermo Haake, Germany) was employed to determine the rheological features of yogurt samples. The rheometer was equipped with a measuring system with a titanium geometry plate having a 40 mm diameter and 1 mm gap. For the determination of the oscillatory rheological attributes, the samples were subjected to frequency dependency experiments conducted at 4 °C from 0.1 to 10.0 Hz. The storage modulus (G′), loss modulus (G″), and complex modulus (G*) were monitored at 1 Hz frequency. The viscosity of the samples was assessed at a constant shear rate of 100 s^−1^.

### 4.10. Sample Extraction

Two grams of yogurt sample added with 20 mL methanol acidified with 0.1% HCl were ultrasonically extracted at ambient temperature for 60 min in an ultrasonic bath (Bandelin Sonorex Digital 10P, Bandelin Electronic GmbH, Berlin, Germany). The extracts were centrifuged at 4000× *g* for 5 min, and the collected supernatants were diluted to 25 mL and filtered through 0.45 μm polyamide membranes. The extraction and filtration were carried out twice for each sample.

### 4.11. Total Phenolic Content, Total Anthocyanin Content, and Radical Scavenging Activity

The total phenolic content was determined in the sample extracts following the Folin–Ciocalteu spectrophotometric method as described by Singleton et al. [80]. For this purpose, 0.1 mL of the prepared extracts and 5 mL of distilled water were accurately pipetted in a 10 mL test tube, followed by 0.5 mL of Folin–Ciocalteu reagent (1:1). After 3 min, 1.5 mL of 20% Na_2_CO_3_ solution and 4.4 mL of distilled water were added, and the mixture was vortexed for 1 min. After standing for 30 min in the dark at 40 °C, the absorbance was read at 765 nm using a UV spectrophotometer (Varian Cary 50, Varian Co., Cary, NC, USA). The results were expressed as mg gallic acid equivalents (GAE) per 100 g yogurt sample.

The total anthocyanin content was determined using the pH differential method proposed by Giusti and Wrolstad [81]. The results were expressed as milligrams of cyanidin-3-glucoside equivalents (CGE) per 100 g yogurt sample.

The radical scavenging activity of the yogurt extracts was evaluated spectrophotometrically according to the DPPH method described by Oliveira et al. [82]. Aliquots of 50 μL extract were mixed in a test tube with 3 mL of 0.004% DPPH solution. After shaking and incubation in the dark for 30 min, the absorbance was measured at 517 nm using a Varian Cary 50 UV spectrophotometer (Varian Co., Palo Alto, CA, USA). The DPPH radical scavenging activity was calculated as follows:

DPPH scavenging activity (%) = (1 − absorbance of sample/absorbance of blank) × 100

Trolox was used as the standard for the calibration curve, and the results were expressed as milimoles Trolox per 100 g of yogurt sample.

### 4.12. Sensory Analysis

The sensory evaluation of yogurt samples was performed one day after enrichment with BPP using a 5-point hedonic scale ranging from 1 = “dislike extremely” to 5 = “like extremely”. The sensory evaluation was carried out by 24 panelists recruited among members of staff and students from the Department of Horticulture and Food Science of the University of Craiova. Panelists were invited to rate their preference for appearance, flavor, taste, consistency, and overall acceptability. The yogurt samples were served in plastic pots at 20 °C. Panelists randomly received the samples identified with aleatory numbers. They were instructed to rinse their palates with bread and water between samples. In order to perform principal component analysis (PCA), XLSTAT 2021.2.1 software was used.

### 4.13. Statistical Analysis

All the assays were performed at least in triplicate, and the results were presented as mean ± standard deviation. The means multiple comparisons were carried out using the one-way analysis of variance (one-way ANOVA), and significant differences (*p* < 0.05) among means were determined by the Fisher’s least significant difference (LSD) multiple range test using Statgraphics Centurion XVI.I software (StatPoint Technologies, Warrenton, VA, USA).

## Figures and Tables

**Table 1:** Proximate composition, titratable acidity, and fatty acid profile of bilberry pomace powder.

Parameter	Content
Dry matter	89.82 ± 0.65 g/100 g
Crude protein	8.26 ± 0.42 g/100 g
Crude fat	8.31 ± 0.43 g/100 g
Crude fiber	11.43 ± 0.37 g/100 g
Ash	1.09 ± 0.23 g/100 g
Titratable acidity	5.45 ± 0.31 g citric acid/100 g
Fatty acids profile	
Saturated fatty acids (SFA)	8.21 ± 0.34 g/100 g fatty acids
Monounsaturated fatty acids (MUFA)	18.64 ± 0.69 g/100 g fatty acids
Polyunsaturated fatty acids (PUFA), of which:	72.86 ± 2.96 g/100 g fatty acids
n-3	38.11 ± 1.39 g/100 g fatty acids
n-6	34.75 ± 1.57 g/100 g fatty acids
n-6/n-3	0.91

**Table 2:** Color parameters of the control and enriched yogurt samples during 28 days of storage.

Storage Time (Days)	Sample	L*	a*	b*	C*	h*	ΔE
Day 1	YC	83.37 ± 3.02 ^dB^	−0.23 ± 0.07 ^aA^	10.75 ± 0.23 ^dB^	10.76 ± 0.23 ^aB^	88.83 ± 0.39 ^cA^	-
	Y0.5%BPP	55.09 ± 2.49 ^cB^	16.57 ± 0.73 ^bB^	−2.91 ± 0.17 ^cA^	16.82 ± 0.75 ^bB^	9.97 ± 0.34 ^aA^	35.69 ± 3.74 ^aA^
	Y1.0%BPP	43.56 ± 1.52 ^b^	18.95 ± 0.43 ^cB^	−3.46 ± 0.20 ^bA^	19.26 ± 0.42 ^cB^	10.35 ± 0.67 ^aA^	46.42 ± 1.53 ^bAB^
	Y1.5%BPP	40.20 ± 0.80 ^aC^	19.26 ± 0.70 ^cB^	−4.16 ± 0.19 ^aA^	19.70 ± 0.71 ^cB^	12.21 ± 0.45 ^bC^	49.68 ± 1.66 ^cA^
Day 14	YC	83.80 ± 1.33 ^dB^	−0.23 ± 0.05 ^aA^	9.05 ± 0.86 ^cA^	9.05 ± 0.86 ^aA^	88.61 ± 0.29 ^cA^	-
	Y0.5%BPP	53.14 ± 0.55 ^cAB^	15.90 ± 0.48 ^bAB^	−2.88 ± 0.17 ^bA^	16.16 ± 0.49 ^bAB^	10.26 ± 0.44 ^abA^	36.64 ± 1.99 ^aA^
	Y1.0%BPP	42.00 ± 1.93 ^bAB^	18.58 ± 0.31 ^cB^	−3.33 ± 0.22 ^abA^	18.88 ± 0.31 ^cB^	10.17 ± 0.65 ^aA^	47.49 ± 1.82 ^bB^
	Y1.5%BPP	31.94 ± 1.67 ^aB^	18.73 ± 0.55 ^cAB^	−3.60 ± 0.11 ^aB^	19.07 ± 0.53 ^cB^	10.89 ± 0.51 ^bB^	56.66 ± 1.81 ^cB^
Day 28	YC	79.87 ± 2.89 ^dA^	−0.21 ± 0.04 ^aA^	8.63 ± 0.47 ^bA^	8.63 ± 0.47 ^aA^	88.68 ± 0.23 ^cA^	-
	Y0.5%BPP	52.86 ± 1.04 ^cA^	15.18 ± 0.27 ^bA^	−2.80 ± 0.14 ^aA^	15.43 ± 0.24 ^bA^	10.46 ± 0.67 ^bA^	33.15 ± 2.40 ^aA^
	Y1.0%BPP	40.37 ± 2.13 ^bA^	17.52 ± 0.75 ^cA^	−2.94 ± 0.05 ^aB^	17.77 ± 0.73 ^cA^	9.54 ± 0.49 ^aA^	44.82 ± 1.25 ^bA^
	Y1.5%BPP	29.53 ± 1.24 ^aA^	18.05 ± 0.33 ^cA^	−2.98 ± 0.15 ^aC^	18.30 ± 0.32 ^cA^	9.37 ± 0.51 ^aA^	54.81 ± 2.91 ^cB^

Note. Different lowercase letters are indicative of significant differences between yogurt formulations (*p* < 0.05) for the same storage period, while different uppercase letters reveal significant differences between sampling times for the same yogurt formulation (*p* < 0.05); YC—control yogurt; Y0.5%BPP—yogurt with 0.5% BPP addition; Y1.0%BPP—yogurt with 1.0% BPP addition; Y1.5%BPP—yogurt with 1.5% BPP addition.

**Table 3:** Physicochemical characteristics of the control and enriched yogurt samples during 28 days of storage.

Storage Time (Days)	Sample	pH	Acidity (% Lactic Acid)	Syneresis (%)	WHC (%)
Day 1	YC	4.70 ± 0.03 ^dC^	0.86 ± 0.05 ^aA^	59.99 ± 0.34 ^bA^	44.40 ± 0.55 ^aC^
	Y0.5%BPP	4.54 ± 0.04 ^cC^	0.93 ± 0.04 ^bA^	55.35 ± 0.41 ^aA^	45.69 ± 1.77 ^aB^
	Y1.0%BPP	4.44 ± 0.03 ^bB^	0.98 ± 0.04 ^cA^	55.58 ± 0.28 ^aA^	47.31 ± 1.03 ^abA^
	Y1.5%BPP	4.27 ± 0.02 ^aB^	1.04 ± 0.05 ^dA^	55.05 ± 0.36 ^aA^	49.24 ± 1.31 ^bB^
Day 14	YC	4.58 ± 0.03 ^dB^	0.94 ± 0.03 ^aB^	61.83 ± 0.24 ^cB^	42.52 ± 0.43 ^aB^
	Y0.5%BPP	4.46 ± 0.02 ^cB^	0.96 ± 0.03 ^bB^	56.28 ± 0.33 ^bB^	45.10 ± 0.32 ^bAB^
	Y1.0%BPP	4.40 ± 0.03 ^bB^	1.02 ± 0.05 ^cB^	56.75 ± 0.26 ^bB^	47.08 ± 0.56 ^cA^
	Y1.5%BPP	4.26 ± 0.02 ^aB^	1.09 ± 0.04 ^d^	55.36 ± 0.28 ^aA^	48.56 ± 0.52 ^dB^
Day 28	YC	4.47 ± 0.04 ^cA^	0.95 ± 0.06 ^aC^	62.95 ± 0.31 ^cC^	40.06 ± 0.38 ^aA^
	Y0.5%BPP	4.37 ± 0.03 ^bA^	1.00 ± 0.03 ^bC^	58.20 ± 0.26 ^bC^	43.48 ± 0.45 ^bA^
	Y1.0%BPP	4.32 ± 0.03 ^bA^	1.02 ± 0.03 ^cC^	58.05 ± 0.19 ^bC^	45.87 ± 0.70 ^cA^
	Y1.5%BPP	4.16 ± 0.03 ^aA^	1.11 ± 0.05 ^dC^	56.77 ± 0.28 ^aB^	47.04 ± 0.61 ^dA^

Note.Different lowercase letters are indicative of significant differences among yogurt formulations (*p* < 0.05) for the same storage period, while different uppercase letters reveal significant differences among sampling times for the same yogurt formulation (*p* < 0.05); YC—control yogurt; Y0.5%BPP—yogurt with 0.5% BPP addition; Y1.0%BPP—yogurt with 1.0% BPP addition; Y1.5%BPP—yogurt with 1.5% BPP addition.

**Table 4:** Texture parameters of the control and enriched yogurt samples during 28 days of storage.

Storage Time (Days)	Sample	Hardness (g)	Adhesiveness (J)	Gumminess (g)	Stickiness (g)	Chewiness (g)	Cohesiveness
Day 1	YC	20.00 ± 1.00 ^aB^	−24.37 ± 3.71 ^dC^	16.13 ± 0.56 ^aA^	−8.01 ± 0.90 ^dC^	16.51 ± 0.50 ^aA^	0.79 ± 0.04 ^aB^
	Y0.5%BPP	23.33 ± 1.53 ^bB^	−42.95 ± 2.91 ^cC^	19.59 ± 0.54 ^bA^	−10.77 ± 0.68 ^cC^	19.63 ± 0.70 ^abA^	0.83 ± 0.03 ^abB^
	Y1.0%BPP	25.33 ± 1.15 ^bB^	−63.46 ± 3.08 ^bC^	22.00 ± 2.94 ^bA^	−13.41 ± 0.51 ^bC^	26.24 ± 1.04 ^bcB^	0.86 ± 0.06 ^bcA^
	Y1.5%BPP	29.00 ± 1.73 ^cB^	−79.79 ± 2.50 ^aC^	26.39 ± 1.20 ^cA^	−14.97 ± 0.96 ^aC^	29.78 ± 2.73 ^dA^	0.92 ± 0.05 ^cB^
Day 14	YC	29.33 ± 1.53 ^aC^	−44.78 ± 1.54 ^dB^	17.63 ± 0.66 ^aB^	−16.84 ± 1.06 ^dB^	19.48 ± 1.33 ^aB^	0.75 ± 0.01 ^aA^
	Y0.5%BPP	33.33 ± 1.15 ^bC^	−64.72 ± 1.45 ^cB^	24.19 ± 1.35 ^bB^	−25.16 ± 1.41 ^cB^	22.15 ± 0.70 ^bB^	0.83 ± 0.01 ^bB^
	Y1.0%BPP	42.33 ± 0.58 ^cC^	−82.48 ± 2.16 ^bB^	26.49 ± 0.89 ^cB^	−34.91 ± 1.17 ^bB^	26.26 ± 1.05 ^cB^	0.85 ± 0.00 ^bA^
	Y1.5%BPP	45.67 ± 1.53 ^dC^	−105.72 ± 3.76 ^aB^	30.04 ± 1.60 ^dB^	−53.48 ± 2.43 ^aB^	38.33 ± 1.07 ^dB^	0.87 ± 0.01 ^cAB^
Day 28	YC	17.33 ± 0.58 ^aA^	−79.38 ± 2.11 ^dA^	15.72 ± 0.55 ^aA^	−30.11 ± 1.58 ^dA^	18.23 ± 0.95 ^aAB^	0.72 ± 0.01 ^aA^
	Y0.5%BPP	20.67 ± 0.58 ^bA^	−95.19 ± 1.18 ^cA^	18.46 ± 1.08 ^bA^	−45.63 ± 1.57 ^cA^	20.52 ± 0.96 ^bA^	0.76 ± 0.01 ^bA^
	Y1.0%BPP	23.67 ± 0.58 ^cA^	−99.75 ± 0.94 ^bA^	20.38 ± 0.86 ^cA^	−51.13 ± 2.40 ^bA^	22.33 ± 0.93 ^bA^	0.78 ± 0.01 ^bA^
	Y1.5%BPP	25.00 ± 1.00 ^dA^	−113.09 ± 1.09 ^aA^	25.27 ± 0.88 ^dA^	−64.99 ± 2.57 ^aA^	37.98 ± 1.61 ^cB^	0.85 ± 0.02 ^cA^

Note. Different lowercase letters are indicative of significant differences between yogurt formulations (*p* < 0.05) for the same storage period, while different uppercase letters reveal significant differences between sampling times for the same yogurt formulation (*p* < 0.05); YC—control yogurt; Y0.5%BPP—yogurt with 0.5% BPP addition; Y1.0%BPP—yogurt with 1.0% BPP addition; Y1.5%BPP—yogurt with 1.5% BPP addition.

**Table 5:** Elastic modulus (G′), loss modulus (G″), complex modulus (|G*|), and dynamic viscosity (η) of the control and enriched yogurt samples during 28 days of storage.

Storage Time (Days)	Sample	G′ (Pa)	G″ (Pa)	|G*| (Pa)	η in Pa·s
Day 1	YC	129.37 ± 0.97 ^aC^	36.18 ± 0.54 ^aC^	134.31 ± 1.04 ^aC^	0.50 ± 0.01 ^aB^
	Y0.5%BPP	185.30 ± 0.82 ^bB^	48.55 ± 1.22 ^bB^	173.05 ± 1.36 ^bB^	0.53 ± 0.01 ^bB^
	Y1.0%BPP	190.09 ± 0.76 ^cB^	52.99 ± 0.89 ^cB^	193.19 ± 0.89 ^cB^	0.61 ± 0.01 ^cC^
	Y1.5%BPP	194.78 ± 0.96 ^dB^	57.42 ± 0.70 ^dB^	202.97 ± 2.23 ^dB^	0.65 ± 0.01 ^dC^
Day 14	YC	120.54 ± 0.73 ^aB^	32.47 ± 0.62 ^aB^	124.81 ± 0.45 ^aB^	0.57 ± 0.00 ^aC^
	Y0.5%BPP	190.49 ± 0.78 ^bC^	53.58 ± 0.72 ^bC^	198.17 ± 0.93 ^bC^	0.58 ± 0.00 ^abC^
	Y1.0%BPP	193.30 ± 1.04 ^cC^	55.41 ± 0.42 ^cC^	201.31 ± 1.09 ^cC^	0.58 ± 0.01 ^abB^
	Y1.5%BPP	195.36 ± 0.72 ^dB^	56.70 ± 0.38 ^dB^	203.49 ± 0.97 ^dB^	0.59 ± 0.01 ^bB^
Day 28	YC	59.42 ± 0.73 ^aA^	17.61 ± 0.46 ^aA^	62.03 ± 0.29 ^aA^	0.34 ± 0.01 ^aA^
	Y0.5%BPP	71.14 ± 0.44 ^bA^	21.30 ± 0.28 ^bA^	74.54 ± 1.25 ^bA^	0.41 ± 0.01 ^bA^
	Y1.0%BPP	97.67 ± 1.05 ^cA^	29.35 ± 1.09 ^cA^	102.29 ± 1.23 ^cA^	0.47 ± 0.01 ^cA^
	Y1.5%BPP	128.30 ± 0.99 ^dA^	39.55 ± 0.79 ^dA^	134.52 ± 1.04 ^dA^	0.51 ± 0.01 ^dA^

Note. Different lowercase letters are indicative of significant differences between yogurt formulations (*p* < 0.05) for the same storage period, while different uppercase letters reveal significant differences between sampling times for the same yogurt formulation (*p* < 0.05); YC—control yogurt; Y0.5%BPP—yogurt with 0.5% BPP addition; Y1.0%BPP—yogurt with 1.0% BPP addition; Y1.5%BPP—yogurt with 1.5% BPP addition.

**Table 6:** Total phenolic content, total anthocyanin content, and DPPH radical scavenging activity of the control and enriched yogurt samples during 28 days of storage.

Storage Time (Days)	Sample	TPC (mg GAE/100 g)	TAC (mg GCE/100 g)	RSA (μmol Trolox/g)
Day 1	YC	9.88 ± 0.37 ^aB^	-	0.62 ± 0.02 ^aA^
	Y0.5%BPP	24.91 ± 0.89 ^bB^	12.48 ± 0.43 ^aB^	0.77 ± 0.03 ^bAB^
	Y1.0%BPP	42.36 ± 1.25 ^cB^	23.62 ± 0.68 ^bB^	0.86 ± 0.03 ^cAB^
	Y1.5%BPP	62.45 ± 1.66 ^dB^	36.77 ± 1.21 ^cB^	0.98 ± 0.04 ^dAB^
Day 14	YC	9.91 ± 0.41 ^aC^	-	0.63 ± 0.02 ^aA^
	Y0.5%BPP	28.64 ± 1.04 ^bC^	14.17 ± 0.86 ^aC^	0.83 ± 0.03 ^bB^
	Y1.0%BPP	45.27 ± 1.21 ^cC^	26.05 ± 1.25 ^bC^	0.95 ± 0.04 ^cB^
	Y1.5%BPP	66.36 ± 1.98 ^dC^	42.71 ± 1.88 ^cC^	1.06 ± 0.04 ^dB^
Day 28	YC	8.68 ± 0.34 ^aA^	-	0.59 ± 0.02 ^aA^
	Y0.5%BPP	22.09 ± 0.82 ^bA^	9.94 ± 0.73 ^aA^	0.71 ± 0.09 ^bA^
	Y1.0%BPP	37.82 ± 1.28 ^cA^	18.19 ± 0.74 ^bA^	0.80 ± 0.04 ^cA^
	Y1.5%BPP	59.55 ± 1.45 ^dA^	28.49 ± 0.85 ^cA^	0.88 ± 0.05 ^dA^

Note. Different lowercase letters are indicative of significant differences between yogurt formulations (*p* < 0.05) for the same storage period, while different uppercase letters reveal significant differences between sampling times for the same yogurt formulation (*p* < 0.05); YC—control yogurt; Y0.5%BPP—yogurt with 0.5% BPP addition; Y1.0%BPP—yogurt with 1.0% BPP addition; Y1.5%BPP—yogurt with 1.5% BPP addition.

**Table 7:** Nutrition facts of the yogurt used in the experiments.

Nutrition Facts	For 100 g
Energy (kcal)	176 kJ/42 kcal
Fat (g)	1.5
Saturated fat (g)	0.9
Carbohydrates (g)	3.9
Sugars (g)	3.9
Protein (g)	3.2

## Data Availability

Data are contained within the article.
